# μ-(2,2′-Bipyrimidine)-bis­[dichlorido­palladium(II)] dimethyl­formamide monosolvate

**DOI:** 10.1107/S1600536812040779

**Published:** 2012-10-20

**Authors:** Cyril Young, Andreas Roodt, Barend C. B. Bezuidenhoudt

**Affiliations:** a Department of Chemistry, University of the Free State, PO Box 339, Bloemfontein, 9300, South Africa

## Abstract

In the title compound, [Pd_2_Cl_4_(C_8_H_6_N_4_)]·C_3_H_7_NO, the two Pd^2+^ cations have a distorted square-planar coordination sphere and are bridged by a bis-bidentate 2,2′-bipyrimidine ligand. Two terminal chloride anions are also bonded to each of the Pd^2+^ cations. The dinuclear complex and the dimethylformamide solvate molecule lie on the inter­section of a twofold rotation axis and a mirror plane, with disorder present in the solvate mol­ecule. There is a slight distortion from the square-planar metal geometry, as indicated by the bite angles of 81.77 (13)° and 91.63 (5)°. The C and O atoms of the solvent mol­ecule are disordered over two sets of sites of equal occupancy.

## Related literature

The title compound is structurally related to the mono-coord­inated species reported by Hudgens *et al.* (1997 ▶). For background literature on homogenous catalyst models, see: Van Leeuwen (2004 ▶); Meij *et al.* (2005 ▶); Otto *et al.* (2003 ▶); Steyn *et al.* (1997 ▶). For related structures, see: Inagaki *et al.* (2007 ▶); Maekawa *et al.* (1994 ▶). The mono-coordinated platinum counterpart was reported by Kawakami *et al.* (2006 ▶). For the synthetic procedure, see: Boyle *et al.* (2004 ▶).
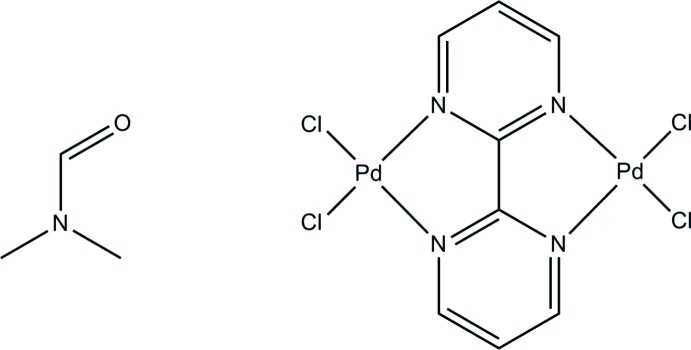


## Experimental

### 

#### Crystal data

[Pd_2_Cl_4_(C_8_H_6_N_4_)]·C_3_H_7_NO
*M* *_r_* = 578.81
Monoclinic, 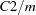

*a* = 10.7299 (6) Å
*b* = 14.2399 (7) Å
*c* = 5.9381 (3) Å
β = 108.229 (2)°
*V* = 861.76 (8) Å^3^
*Z* = 2
Mo *K*α radiation
μ = 2.71 mm^−1^
*T* = 100 K
0.09 × 0.09 × 0.08 mm

#### Data collection

Bruker APEXII diffractometer
Absorption correction: multi-scan (*SADABS*; Bruker, 2008) ▶ *T*_min_ = 0.785, *T*_max_ = 0.814
6102 measured reflections
1108 independent reflections
975 reflections with *I* > 2σ(*I*)
*R*_int_ = 0.041

#### Refinement

*R*[*F* ^2^ > 2σ(*F* ^2^)] = 0.028
*wR*(*F* ^2^) = 0.059
*S* = 1.11
1108 reflections
68 parameters
H-atom parameters constrained
Δρ_max_ = 0.75 e Å^−3^
Δρ_min_ = −0.79 e Å^−3^

### 

Data collection: *APEX2* (Bruker, 2008) ▶; cell refinement: *SAINT-Plus* (Bruker, 2008 ▶); data reduction: *SAINT-Plus*; program(s) used to solve structure: *SHELXS97* (Sheldrick, 2008 ▶); program(s) used to refine structure: *SHELXL97* (Sheldrick, 2008 ▶); molecular graphics: *DIAMOND* (Brandenburg & Putz, 2005) ▶; software used to prepare material for publication: *WinGX* (Farrugia, 1999 ▶).

## Supplementary Material

Crystal structure: contains datablock(s) global, I. DOI: 10.1107/S1600536812040779/gg2102sup1.cif

Structure factors: contains datablock(s) I. DOI: 10.1107/S1600536812040779/gg2102Isup2.hkl

Additional supplementary materials:  crystallographic information; 3D view; checkCIF report

## Figures and Tables

**Fig. 1.**
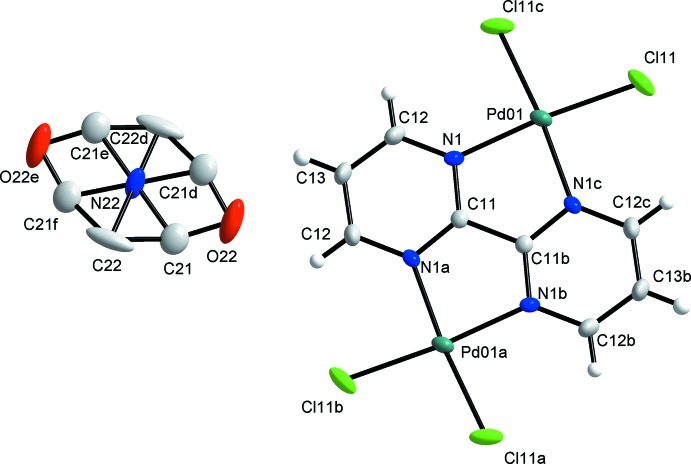
Diamond representation of the title compound, showing the numbering scheme and displacement ellipsoids (50% probability).

## supplementary crystallographic information

### Comment

Although a wide variety of metals are used in catalysis today, the platinum group metals show the most promising catalytic properties (Van Leeuwen (2004). Unfortunately, knowledge surrounding the actual catalysis process on a molecular level is minimal. Various platinum group metals which find application in heterogeneous catalysis are dispersed onto supports with little control. Consequently, this study was undertaken to explore the possibility of using bridging ligands ensure that metals are well dispersed in a controllable fashion. The insight gained by exploring bridged platinum group metals could contribute to ongoing homogeneous catalyst models of the metal complex. (Meij *et al.* (2005), Otto *et al.* (2003) Steyn *et al. (1997)*).

The compound crystallizes in a monoclinic *C*2/*m* space group with *Z* = 2. Both palladium atoms are situated on a twofold rotation axis and three carbon atoms, namely C11, C13 and C22 lie on a mirror plane. O22 of the DMF solvate molecule is situated on a twofold rotation axis and N22 on both a mirror plane and a rotation axis, which essentially gives N22 an occupation of 25%. As a result of the symmetry in the molecule there are only twelve atoms, including the hydrogen atoms, in the asymmetric unit. The geometry of the palladium centers is slightly distorted from the square planar geometry. This is illustrated by the N1—Pd01—N1c and Cl11—Pd01—Cl11*c* angles which are 81.77 (13)° and 91.63 (5)° respectively. The bond lengths and angles for the title compound are comparable to those in literature (Inagaki *et al.* (2007), Maekawa *et al.* (1994)). The palladium-nitrogen bonds of 2.05 Å are marginally longer than the mono-coordinated palladium complex (Hudgens *et al.* (1997)) which has a bond length of 1.99 Å. The Pd—Pd intra-molecular bond distances of 5.47 Å, is slightly shorter than the 5.62 Å for the platinum counterpart (Kawakami *et al.* (2006)).

### Experimental

The title compound was prepared by the modification of the published procedure by Boyle *et al.* (2004). PdCl_2_ (0.200 g, 1,13 *x* 10 ^-3^ mol) was dissolved in boiling acetonitrile. 2,2-Bipyrimidine (0.092 g, 5.71 *x* 10 ^-4^ mol) was added to the solution. Upon addition a yellow precipitate formed. Yield: 0.244 g (85%). ^1^H NMR ((CD_3_)_2_SO): δ 7.9 (t, J = 5.2 Hz, 2H), 9.3 (dd, J = 5.1 Hz, 4H). IR (ATR): 1589 (v_C—H_),1137 (v_C—N_), 813 (v_Ar—H_).

### Refinement

The aromatic, methine, and methyl H atoms were placed in geometrically idealized positions (C—H = 0.93–0.98) and constrained to ride on their parent atoms with *U*_ι__so_ (H) = 1.2*U_eq_*(C) for the aromatic protons. The highest residual electron density was located 0.55 Å from C13 and was essentially meaningless.

### Crystal data

**Table:**

[Pd_2_Cl_4_(C_8_H_6_N_4_)]·C_3_H_7_NO	*F*(000) = 550
*M**_r_* = 578.81	*D*_x_ = 2.231 Mg m^−^^3^
Monoclinic, *C*2/*m*	Mo *K*α radiation, λ = 0.71073 Å
Hall symbol: -C 2y	Cell parameters from 1958 reflections
*a* = 10.7299 (6) Å	θ = 2.9–27.8°
*b* = 14.2399 (7) Å	µ = 2.71 mm^−^^1^
*c* = 5.9381 (3) Å	*T* = 100 K
β = 108.229 (2)°	Cuboid, red
*V* = 861.76 (8) Å^3^	0.09 × 0.09 × 0.08 mm
*Z* = 2	

### Data collection

**Table:**

Bruker APEXII diffractometer	1108 independent reflections
Radiation source: sealed tube	975 reflections with *I* > 2σ(*I*)
Graphite monochromator	*R*_int_ = 0.041
phi and ω scans	θ_max_ = 28.3°, θ_min_ = 3.6°
Absorption correction: multi-scan (*SADABS*; Bruker, 2008)	*h* = −12→14
*T*_min_ = 0.785, *T*_max_ = 0.814	*k* = −18→18
6102 measured reflections	*l* = −7→7

### Refinement

**Table:**

Refinement on *F*^2^	Primary atom site location: structure-invariant direct methods
Least-squares matrix: full	Secondary atom site location: difference Fourier map
*R*[*F*^2^ > 2σ(*F*^2^)] = 0.028	Hydrogen site location: inferred from neighbouring sites
*wR*(*F*^2^) = 0.059	H-atom parameters constrained
*S* = 1.11	*w* = 1/[σ^2^(*F*_o_^2^) + (0.0172*P*)^2^ + 2.5414*P*] where *P* = (*F*_o_^2^ + 2*F*_c_^2^)/3
1108 reflections	(Δ/σ)_max_ < 0.001
68 parameters	Δρ_max_ = 0.75 e Å^−^^3^
0 restraints	Δρ_min_ = −0.79 e Å^−^^3^

### Special details

**Table:**

Geometry. All s.u.'s (except the s.u. in the dihedral angle between two l.s. planes) are estimated using the full covariance matrix. The cell s.u.'s are taken into account individually in the estimation of s.u.'s in distances, angles and torsion angles; correlations between s.u.'s in cell parameters are only used when they are defined by crystal symmetry. An approximate (isotropic) treatment of cell s.u.'s is used for estimating s.u.'s involving l.s. planes.
Refinement. Refinement of *F*^2^ against ALL reflections. The weighted *R*-factor *wR* and goodness of fit *S* are based on *F*^2^, conventional *R*-factors *R* are based on *F*, with *F* set to zero for negative *F*^2^. The threshold expression of *F*^2^ > 2σ(*F*^2^) is used only for calculating *R*-factors(gt) *etc*. and is not relevant to the choice of reflections for refinement. *R*-factors based on *F*^2^ are statistically about twice as large as those based on *F*, and *R*- factors based on ALL data will be even larger.

### Fractional atomic coordinates and isotropic or equivalent isotropic displacement parameters (Å^2^)

**Table:**

	*x*	*y*	*z*	*U*_iso_*/*U*_eq_	Occ. (<1)
C11	0.5	0.4487 (3)	0.5	0.0149 (8)	
C12	0.5694 (3)	0.3120 (2)	0.6987 (6)	0.0270 (8)	
H12	0.6167	0.2793	0.8341	0.032*	
C13	0.5	0.2637 (3)	0.5	0.0370 (13)	
H13	0.5	0.1984	0.5	0.044*	
N1	0.5696 (2)	0.40577 (17)	0.6995 (4)	0.0152 (5)	
Cl11	0.72957 (8)	0.61422 (6)	1.23998 (14)	0.0303 (2)	
Pd01	0.65034 (3)	0.5	0.96630 (6)	0.01736 (12)	
N22	0.5	0	0	0.0288 (14)	
O22	0.5	0.1536 (5)	0	0.052 (2)	0.5
C21	0.4476 (8)	0.0853 (5)	−0.1298 (13)	0.0356 (18)	0.5
C22	0.4325 (11)	0	−0.2451 (19)	0.053 (4)	0.5

### Atomic displacement parameters (Å^2^)

**Table:**

	*U*^11^	*U*^22^	*U*^33^	*U*^12^	*U*^13^	*U*^23^
C11	0.013 (2)	0.015 (2)	0.018 (2)	0	0.0063 (17)	0
C12	0.0231 (18)	0.0158 (15)	0.035 (2)	0.0002 (13)	−0.0007 (15)	0.0069 (13)
C13	0.034 (3)	0.012 (2)	0.051 (3)	0	−0.008 (3)	0
N1	0.0112 (12)	0.0180 (12)	0.0155 (13)	0.0008 (10)	0.0027 (10)	0.0040 (10)
Cl11	0.0220 (4)	0.0488 (5)	0.0189 (4)	−0.0109 (4)	0.0045 (3)	−0.0137 (4)
Pd01	0.01263 (19)	0.0256 (2)	0.01300 (18)	0	0.00278 (13)	0
N22	0.052 (4)	0.016 (3)	0.026 (3)	0	0.025 (3)	0
O22	0.095 (7)	0.019 (4)	0.064 (6)	0	0.058 (6)	0
C21	0.037 (5)	0.038 (4)	0.041 (4)	−0.001 (3)	0.025 (4)	0.002 (4)
C22	0.015 (5)	0.123 (12)	0.018 (5)	0	0.003 (4)	0

### Geometric parameters (Å, º)

**Table:**

C11—N1^i^	1.335 (3)	N22—C22	1.408 (11)
C11—N1	1.335 (3)	N22—C22^iv^	1.408 (11)
C11—C11^ii^	1.462 (8)	N22—C21^v^	1.454 (8)
C12—N1	1.336 (4)	N22—C21^vi^	1.454 (8)
C12—C13	1.368 (4)	N22—C21	1.454 (8)
C12—H12	0.93	N22—C21^iv^	1.454 (8)
C13—C12^i^	1.368 (4)	O22—C21	1.258 (9)
C13—H13	0.93	O22—C21^v^	1.258 (9)
N1—Pd01	2.050 (2)	C21—C22	1.379 (9)
Cl11—Pd01	2.2682 (8)	C21—C21^v^	1.598 (16)
Pd01—N1^iii^	2.050 (2)	C22—C21^vi^	1.379 (9)
Pd01—Cl11^iii^	2.2682 (8)		
			
N1^i^—C11—N1	125.5 (4)	C22^iv^—N22—C21^vi^	122.4 (3)
N1^i^—C11—C11^ii^	117.24 (18)	C21^v^—N22—C21^vi^	180.0 (3)
N1—C11—C11^ii^	117.24 (18)	C22—N22—C21	57.6 (3)
N1—C12—C13	120.4 (3)	C22^iv^—N22—C21	122.4 (3)
N1—C12—H12	119.8	C21^v^—N22—C21	66.7 (6)
C13—C12—H12	119.8	C21^vi^—N22—C21	113.3 (6)
C12—C13—C12^i^	119.6 (4)	C22—N22—C21^iv^	122.4 (3)
C12—C13—H13	120.2	C22^iv^—N22—C21^iv^	57.6 (3)
C12^i^—C13—H13	120.2	C21^v^—N22—C21^iv^	113.3 (6)
C11—N1—C12	117.1 (3)	C21^vi^—N22—C21^iv^	66.7 (6)
C11—N1—Pd01	111.7 (2)	C21—N22—C21^iv^	180.0 (6)
C12—N1—Pd01	131.1 (2)	C21—O22—C21^v^	78.9 (8)
N1—Pd01—N1^iii^	81.77 (13)	O22—C21—C22	160.9 (9)
N1—Pd01—Cl11	174.98 (7)	O22—C21—N22	107.2 (6)
N1^iii^—Pd01—Cl11	93.29 (7)	C22—C21—N22	59.5 (5)
N1—Pd01—Cl11^iii^	93.29 (7)	O22—C21—C21^v^	50.6 (4)
N1^iii^—Pd01—Cl11^iii^	174.98 (7)	C22—C21—C21^v^	114.6 (6)
Cl11—Pd01—Cl11^iii^	91.63 (5)	N22—C21—C21^v^	56.7 (3)
C22—N22—C22^iv^	180.0000 (10)	C21—C22—C21^vi^	123.5 (10)
C22—N22—C21^v^	122.4 (3)	C21—C22—N22	62.9 (5)
C22^iv^—N22—C21^v^	57.6 (3)	C21^vi^—C22—N22	62.9 (5)
C22—N22—C21^vi^	57.6 (3)		

Symmetry codes: (i) −*x*+1, *y*, −*z*+1; (ii) −*x*+1, −*y*+1, −*z*+1; (iii) *x*, −*y*+1, *z*; (iv) −*x*+1, −*y*, −*z*; (v) −*x*+1, *y*, −*z*; (vi) *x*, −*y*, *z*.
